# Accumulation of F-actin drives brain aging and limits healthspan in *Drosophila*

**DOI:** 10.21203/rs.3.rs-3158290/v1

**Published:** 2023-08-01

**Authors:** Edward T. Schmid, Joseph M. Schinaman, Kylie S. Williams, David W. Walker

**Affiliations:** 1 Department of Integrative Biology and Physiology, University of California, Los Angeles, Los Angeles, California 90095, USA.; 2 Molecular Biology Institute, University of California, Los Angeles, Los Angeles, California 90095, USA.

**Keywords:** Lifespan, Mitophagy, Fhos, proteostasis

## Abstract

The actin cytoskeleton is a key determinant of cell and tissue homeostasis. However, tissue-specific roles for actin dynamics in aging, notably brain aging, are not understood. Here, we show that there is an age-related increase in filamentous actin (F-actin) in *Drosophila* brains, which is counteracted by prolongevity interventions. Critically, modulating F-actin levels in aging neurons prevents age-onset cognitive decline and extends organismal healthspan. Mechanistically, we show that autophagy, a recycling process required for neuronal homeostasis, is disabled upon actin dysregulation in the aged brain. Remarkably, disrupting actin polymerization in aged animals with cytoskeletal drugs restores brain autophagy to youthful levels and reverses cellular hallmarks of brain aging. Finally, reducing F-actin levels in aging neurons slows brain aging and promotes healthspan in an autophagy-dependent manner. Our data identify excess actin polymerization as a hallmark of brain aging, which can be targeted to reverse brain aging phenotypes and prolong healthspan.

## Introduction

The actin cytoskeleton dictates the shape and polarity of a cell and is essential in numerous diverse fundamental processes including cellular division, motility, phagocytosis, organelle trafficking, and signaling ^1^. Actin can be found in two forms: monomeric (G-actin) and filamentous (F-actin). Assembly and disassembly of actin filaments are regulated by a large number of actin-interacting proteins ^2^, making maintenance of the actin cytoskeleton highly susceptible to disruption caused by aging. In fact, aging has been found to cause not only changes in the expression of actin genes but also disruption of the organization and dynamics of the actin cytoskeleton ^3^. More specifically, recent studies in *C. elegans* have shown that the organization of the actin cytoskeleton deteriorates in the hypodermis, muscles, and intestines during aging ^4,5^. Critically, however, the interplay between actin dynamics and neuronal aging has not been characterized in any species. Intriguingly, F-actin-rich paracrystalline inclusions (known as Hirano bodies) have been described in aging and neurodegeneration, including Alzheimer’s disease and other tauopathies ^6–12^. Importantly, animal models expressing mutant actin binding proteins suggest Hirano bodies are associated with impaired synaptic responses and decreased spatial working memory ^13,14^. Furthermore, tau-induced neurodegeneration is associated with accumulation of F-actin and the formation of actin-rich rods in both *Drosophila* and mouse models ^15^. Critically, reducing F-actin levels leads to modification of tau- and other models of neurodegeneration in *Drosophila* ^15–17^. Hence, it is apparent that excess actin polymerization and actin-rich inclusions play a causal role in neurotoxicity in the context of certain diseases of the brain ^15,18,19^. However, despite considerable focus upon identifying key hallmarks of aging ^20^, including brain aging ^21^, the role of actin dynamics in the aging brain has not been characterized.

Two of the best characterized hallmarks of aging are the accumulation of damaged proteins and dysfunctional mitochondrial ^20^. Macroautophagy (hereafter, autophagy) is a fundamental process by which cellular waste (referred to as autophagic cargo), including nucleic acids, proteins, lipids and organelles, is isolated inside specialized vesicles called autophagosomes for recycling via lysosome-mediated degradation ^22^. A growing body of evidence indicates that autophagic activity declines with age ^23^, including in the aged brain ^24–26^. This age-related decline has also been reported in the context of mitochondrial autophagy (mitophagy), a cargo-specific form of autophagy that degrades dysfunctional mitochondria ^27–29^. Importantly, stimulating autophagy or mitophagy in aging neurons can prolong lifespan in model organisms ^20,29–32^. Hence, there is an emerging understanding that disabled autophagy/mitophagy contributes to brain aging and, thereby, limits lifespan ^20,23,30^. The age-associated dysregulation of autophagy has been demonstrated by the accumulation of autophagosomes, possibly due to impaired lysosomal fusion and/or degradation, yet the underlying mechanisms are not understood ^23^. Actin cytoskeleton dynamics play important roles throughout the various steps of autophagy ^33^. Indeed, studies in cell culture have shown that actin dynamics regulates lysosome-autophagosome fusion ^34,35^, including by promoting autophagosome trafficking to the lysosome ^36^. Interestingly, recent work has shown that excess F-actin stabilization can disrupt autophagic activity in a *Drosophila* Parkinson’s disease model of α-synuclein neurotoxicity ^37^ . However, the interplay between actin dynamics, autophagy and brain aging remains unexplored.

In this study, we set out to examine the role of actin dynamics in brain aging. Using the *Drosophila* model, we have identified a striking increase in F-actin in the aged brain concomitant with the formation of actin-rich rods not present in young animals. Furthermore, we have found that F-actin levels in the brain correlate with the health of aged animals. Flies undergoing dietary restriction or treated with rapamycin, two evolutionarily conserved approaches to lifespan extension ^38,39^, both show a reduction in F-actin in aged brains. To establish causal relationships, we have identified multiple interventions targeting neuronal actin dynamics that can slow brain aging and prolong healthspan. More specifically, we show that adult-onset, neuron-specific inhibition of *Fhos* (*Formin homology 2 domain containing ortholog*), a FHOD class formin that nucleates actin filaments ^40^, improves cognitive function in aged flies and dramatically improves multiple markers of organismal healthspan. Using both genetic and pharmacological approaches, we show that excess F-actin polymerization leads to impaired autophagic activity and the accumulation of dysfunctional mitochondria in the aged brain. Remarkably, we show that treating aged animals with cytoskeletal drugs, to disrupt actin polymerization, can reverse age-onset impairments in brain autophagy and improve cognitive performance. Finally, we show that improvements in autophagy in the aged brain are necessary for the beneficial effects of neuronal F-actin modulation. Together, our findings reveal neuronal dysregulation of actin dynamics as a novel hallmark of aging, which can be targeted to restore autophagic activity, improve brain function and prolong healthspan.

## Results

### F-actin accumulates in aging *Drosophila* brains and correlates with aging health

**Extended Data Figure 1.**
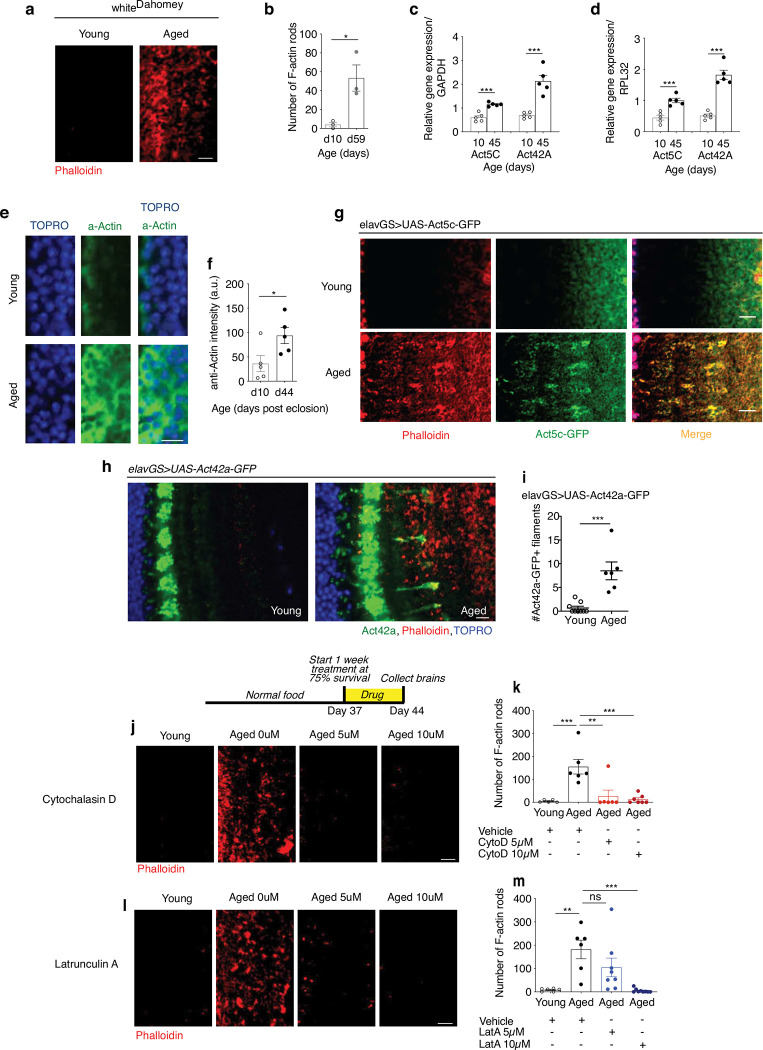
Actin polymerization increases in the aging *Drosophila* brain. (a) Immunostaining of brains at 63x magnification from young (10-day-old) and aged (59-day-old) white Dahomey flies, showing actin-rich rods (red channel, phalloidin). Scale bar is 5 μm. (b) Quantification of actin-rich rods by phalloidin staining per 1mm^2^ area of brain optic lobes as shown in (a). n = 3 flies per condition. *p = 0.0249; unpaired t-test. (c) qPCR analysis of *Act5c* and *Act42a* levels relative to *GAPDH* in fly heads on days 10 and 45 post eclosion from Canton S flies. n = 5 biological replicates with 5 dissected heads pooled per replicate. ***p (*Act5c*) = 0.0002; ***p (*Act42a*) = 0.0004, unpaired t-tests. (d) qPCR analysis of *Act5c* and *Act42a* levels relative to *RPL32* in fly heads on days 10 and 45 post eclosion from Canton S flies. n = 5 biological replicates with 5 dissected heads pooled per replicate. ***p (*Act5c*) = 0.0009; ***p (*Act42a*) < 0.0001, unpaired t-tests. (e) Immunostaining of brains at 63x magnification from young (10-day-old) and aged (45-day-old) Canton S flies, showing anti-actin (green channel) and nuclear DNA (blue channel, To-Pro-3). Scale bar is 5 μm. (f) Quantification of actin intensity by anti-actin antibody in brains as shown in (e). n = 5 lies per condition. *p = 0.0399, unpaired t-test. (g) Immunostaining of brains at 63x magnification from young (10-day-old) and aged (30-day-old) *elavGS*>*UAS-Act5c-GFP* flies, showing F-actin intensity (red channel, Phalloidin) and Act5c-GFP (green channel). Scale bar is 5 μm. (h) Immunostaining of brains at 63x magnification from young (10-day-old) and aged (45-day-old) *elavGS*>*UAS-Act42a-GFP* flies, showing F-actin (red channel, phalloidin), Act42a-GFP (green channel), and nuclear DNA (blue channel, To-Pro-3). Scale bar is 5 μm. (i) Quantification of Act42a-GFP filaments observed in brain areas as shown in (h). n = 6–9 flies per condition, as indicated. ***p = 0.0006, unpaired t-test. (j) Immunostaining of brains at 63x magnification from young (10-day-old) and aged (45-day-old) Canton S flies given vehicle (DMSO), 5μM cytochalasin D, or 10μM cytochalasin D as indicated from days 37–44 post eclosion, showing actin-rich rods in brain optic lobes (red channel, phalloidin). Scale bar is 5 μm. Accompanying diagram indicates drug feeding paradigm. (k) Quantification actin-rich rods by phalloidin stain per 1mm2 area of brains as shown in (j). n = 5–7 flies per condition, as indicated. **p = 0.0018, ***p (young vs. aged + vehicle) = 0.0006, ***p (aged + vehicle vs. aged + 10 μM cytochalasin D) = 0.0004; one-way ANOVA, Tukey’s multiple comparisons test. (l) Immunostaining of brains at 63x magnification from young (10-day-old) and aged (45-day-old) Canton S flies given vehicle (DMSO), 5μM Latrunculin A, or 10μM Latrunculin A as indicated from days 37–44 post eclosion, showing F-actin accumulation (red channel, Phalloidin). Scale bar is 5 μm. (m) Quantification of the number of actin-rich rods by phalloidin stain per 1mm^2^ area of brains as shown in (l). n = 6–9 flies per condition, as indicated. ns = non-significant, **p = 0.0022, ***p = 0.0007; one-way ANOVA, Tukey’s multiple comparisons test.

**Figure 1.**
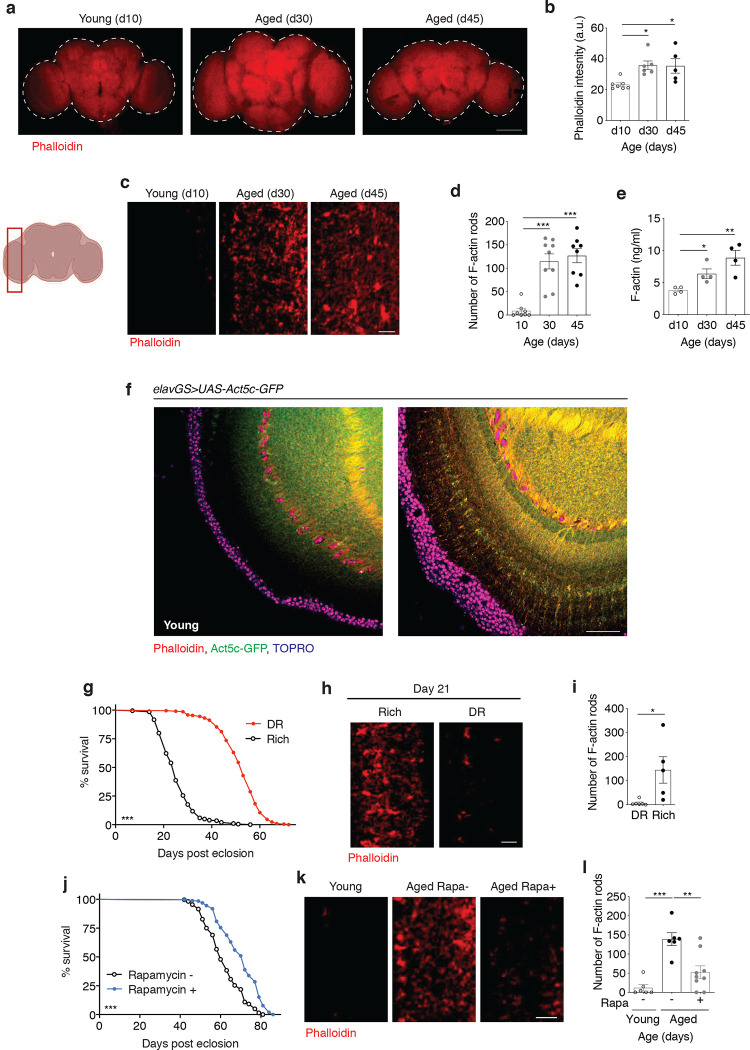
F-actin accumulates in aged *Drosophila* brains and correlates with health. (a) Immunostaining of brains at 10x magnification from young (10-day-old) and aged (30-day-old and 45-day-old, as indicated) Canton S flies, showing F-actin fluorescence intensity (red channel, phalloidin). Scale bar is 100 μm. (b) Quantification of mean phalloidin fluorescence intensity in brains as shown in (a). n = 5–7 flies per condition, as indicated. *p (d10 vs. d30) = 0.0158, * (d10 vs. d45) p=0.0263; one-way ANOVA, Tukey’s multiple comparisons test. (c) Immunostaining of brains at 63x magnification from young (10-day-old) and aged (30-day-old and 45-day-old, as indicated) Canton S flies, showing actin-rich rods (red channel, Phalloidin). Scale bar is 5 μm. Accompanying diagram indicates brain region where imaging was conducted. (d) Quantification of actin-rich rods by phalloidin staining per 1mm^2^ area of brain optic lobes as shown in (c). n = 8–9 flies per condition, as indicated. ***p (d10 vs d30 and d10 vs d45) < 0.0001; one-way ANOVA, Tukey’s multiple comparisons test. (e) Quantification of F-actin protein by ELISA using head homogenates from young (10-day-old) and aged (30-day-old and 45-day-old, as indicated) Canton S flies. n = 4 homogenates generated with 5 brains each per condition. *p = 0.0192, **p = 0.0053, unpaired t-tests. (f) Immunostaining of brains at 63x magnification from young (10-day-old) and aged (30-day-old) *elavGS*>*UAS-Act5c-GFP* flies, showing F-actin (red channel, phalloidin), Act5c-GFP (green channel), and nuclear DNA (blue channel, To-Pro-3). Scale bar is 20 μm. (g) Survival curves of Canton S flies given a rich diet (5.0% yeast extract) versus those undergoing dietary restriction (DR, 0.5% yeast extract) from day 4 post eclosion onwards. ***p=0.001; log-rank test; n > 140 flies. (h) Immunostaining of brains at 63x magnification from Canton S flies aged day 21 post eclosion provided a rich or restricted (DR) diet, as in (g), showing actin-rich rods (red channel, phalloidin). Scale bar is 5 μm. (i) Quantification of actin-rich rods by phalloidin stain per 2 mm^2^ area of brains as shown in (C). n = 5–6 flies per condition, as indicated. *p =0.0225, unpaired t-test. (j) Survival curves of white Dahomey flies given 10uM Rapamycin or vehicle from day 4 post eclosion onwards. ***p<0.0001; log-rank test; n > 250 flies per condition. (h) Immunostaining of brains at 63x magnification from young (10-day-old) and aged (45-day-old) white Dahomey flies given10uM Rapamycin or vehicle, showing actin-rich rods (red channel, phalloidin). Scale bar is 5 μm. (l) Quantification of actin-rich rods by phalloidin stain per 2 mm^2^ area of brains as shown in (k). n = 6–9 flies per condition, as indicated. **p = 0.0023., ***p = 0.0001; one-way ANOVA, Tukey’s multiple comparisons test.

Recent work in *C. elegans* has shown that non-neuronal tissues are associated with the destabilization of actin filaments and a decline in cytoskeletal integrity with age ^5^. However, little is known about changes to actin dynamics in the brains of aging organisms. In this study, we set out to examine brain actin dynamics in naturally aging animals using the *Drosophila* model. Using immunofluorescent microscopy (IF), we began by comparing wild type fly brains collected from young flies (10 days) to those isolated from middle-age (30 days) and late-age (45 days) flies using phalloidin to stain for F-actin^15,17,37,41^. Remarkably, we detected a significant increase in total F-actin levels when comparing young brains to middle-age and late-age brains (Fig 1a,b). At increased magnification, we observed F-actin-rich rods accumulating in the aging brain that were absent in young brains (Fig 1c,d). These observations were corroborated with an additional control laboratory strain (Extended Data Fig 1a,b). To further validate these findings, we collected protein homogenates from the heads of young and aged flies for enzyme-linked immunosorbent assays (ELISAs). Both middle-age and later-aged fly heads had significantly more F-actin compared to young controls (Fig 1e). Interestingly, both cytoplasmic actin isoforms expressed in *Drosophila* neurons, *Act5c* and *Act42a 42*, were observed to increase transcriptionally by quantitative polymerase chain reaction (qPCR) in aged head samples when compared to two other genes considered to be expressed at a steady state: *GAPDH* and *RPL32* (Extended Data Fig 1c,d). Although actin genes are widely considered to be ‘housekeeping genes’, these findings argue that actin expression is dynamic with age in *Drosophila* brains.

To further extend our observations of increased F-actin polymerization in aged fly brains, we used reporter lines expressing Act5c and Act42a tagged with green fluorescent protein (GFP). Flies expressing Act5c-GFP in neurons showed an increase in Act5c with age that colocalized with phalloidin staining (Fig 1f, Extended Data Fig 1e). Neuronal expression of Act42a-GFP showed actin-rich structures in aging brains that were absent in young brains (Extended Data Fig f,g). Interestingly, the distribution of Act42a-GFP in aging *Drosophila* brains showed a pattern distinct from that of Act5c-GFP and phalloidin, suggesting a different distribution of this actin isoform in neurons. Additionally, labeling brains with anti-actin antibody revealed a greater overall actin intensity in aged brains compared to young brains (Extended Data Fig h,i). The pharmacological reagents cytochalasin D and latrunculin A are commonly used to depolymerize actin filaments ^43,44^. Feeding aged flies cytochalasin D (Extended Data Fig j,k) or latrunculin A (Extended Data Fig 1,m) for one week was sufficient to ablate the age-associated accumulation of actin-rich rods in brains. Cumulatively, these observations support a model in which F-actin polymerization increases in *Drosophila* brains with age.

To assess if F-actin polymerization in aged brains was reflective of aging health or if it occurred universally with chronological age, we assessed flies from two widely studied lifespan extension strategies. Dietary restriction (DR) and/or protein restriction is an evolutionarily conserved approach to slow aging and promote lifespan ^45^. Flies fed a low protein diet had a significantly longer lifespan compared to flies provided a high-protein diet (Fig 1g). Using IF, we observed actin-rich rods in the brains of flies on a rich diet at young middle-age (21 days post-eclosion) that were absent in the brains of flies undergoing DR (Fig 1h,i). We next tested the effect of treating flies with rapamycin, a small molecule that has also been shown to prolong lifespan in evolutionarily diverse species via inhibition mTORC1 ^39^. Consistent with previous observations ^46,47^, feeding flies rapamycin significantly extended their lifespan compared to vehicle-fed controls (Fig 1j). Remarkably, aged flies fed rapamycin had significantly fewer actin-rich rods in the brain compared to age-matched controls (Fig 1k,l). Together, these findings suggest that age-associated F-actin polymerization in *Drosophila* brains reflects aging health and can be delayed using prolongevity strategies.

### Genetic targeting of neuronal F-actin extends organismal healthspan and lifespan

**Extended Data Figure 2.**
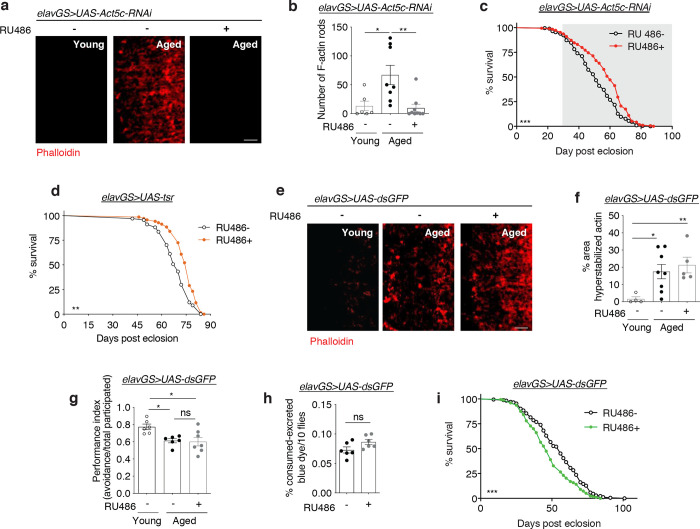
Neuronal knockdown of actin genes, and not *GFP*, reduces age-associated actin-rich rods in the brain and extends organismal lifespan. (a) Immunostaining brains at 63x magnification from young (10-day-old) and aged (45-day-old) *elavGS*>*UAS-Act5c-RNAi* flies with or without RU486-mediated transgene expression from day 5 onward, showing actin-rich rods (red channel, phalloidin). Scale bar is 5 μm. (b) Quantification of actin-rich rods in brains as shown in (a). n = 6–9 flies per condition, as indicated. *p = 0.0160, **p = 0.0045, one-way ANOVA, Tukey’s multiple comparisons test. (c) Survival curve of *elavGS*>*UAS-Act5c-RNAi* flies with or without RU486-mediated transgene expression from day 5 onward. ***p = <0.0001, log-rank test. n = 197 RU− and 189 RU+ biologically independent animals. RU486 or vehicle was provided in the media at a concentration of 25 ug/ml in the indicated treatment group. (d) Survival curve of *elavGS*>*UAS-tsr* flies with or without RU486-mediated transgene expression from day 5 onward. **p = 0.0027, log-rank test. n = 136 RU− and RU+ biologically independent animals. RU486 or vehicle was provided in the media at a concentration of 50 ug/ml in the indicated treatment group. (e) Immunostaining brains at 63x magnification from young (10-day-old) and aged (45-day-old) *elavGS*>*UAS-dsGFP* flies with or without RU486-mediated transgene expression from day 5 onward, showing actin-rich rods (red channel, phalloidin). Scale bar is 5 μm. (f) Quantification of actin-rich rods in brains as shown in (d). n = 4–8 flies per condition, as indicated. *p = 0.0259, **p = 0.0075, unpaired t-tests. (g) Performance index in olfactory aversion training in 37-day-old *elavGS*>*UAS-dsGFP* flies with or without RU486-mediated transgene expression from day 5 onward, assessed by the number of flies avoiding a shock-associated scent versus the total number of flies participating in the assay. ns = non-significant, *p (young vs aged RU−) = 0.0294, *p (young vs. aged RU+) = 0.0162, one-way ANOVA, Tukey’s multiple comparisons test. (h) Con-ex feeding assay of 10-day-old *elavGS*>*UAS-dsGFP* flies with or without RU486-mediated transgene expression from day 5 onward. n = 6 vials of 10 flies per condition. ns = nonsignificant, unpaired t-test. (i) Survival curve of *elavGS*>*UAS-dsGFP* flies with or without RU486-mediated transgene expression from day 5 onward. ***p = 0.0002, log-rank test. n = 173 RU− and 177 RU+ biologically independent animals. RU486 or vehicle was provided in the media at a concentration of 50 ug/ml in the indicated treatment groups. Data are presented as scatter plots overlaying mean values +/− SEM.

**Figure 2.**
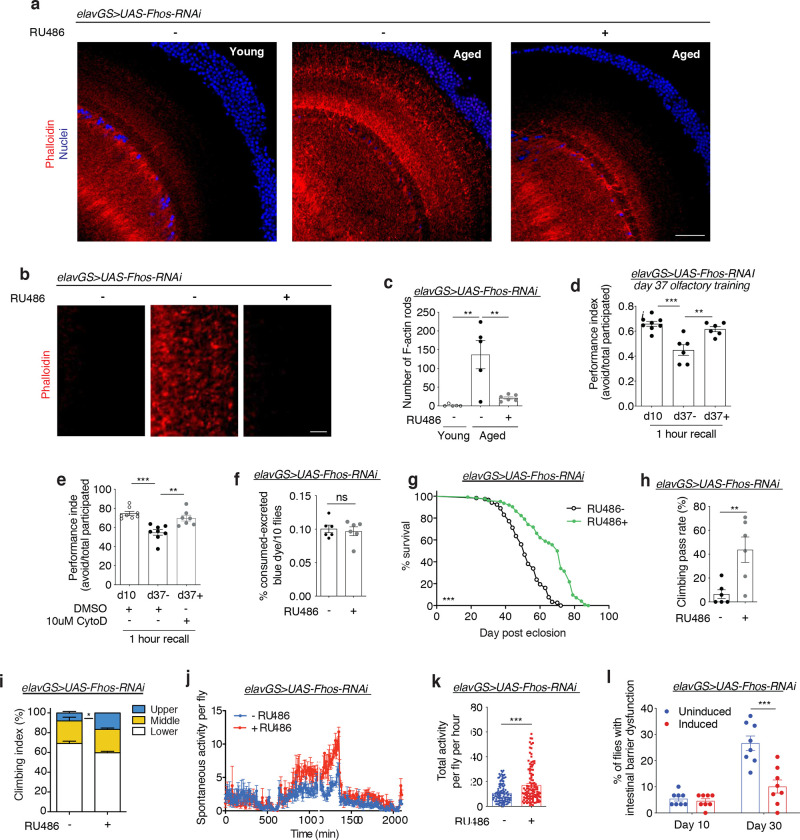
Neuronal knockdown of *Fhos* reduces age-associated actin-rich rods and extends healthspan. (a) Immunostaining of optic lobes at 63x magnification from young (10-day-old) and aged (45-day-old) *elavGS*>*UAS-Fhos-RNAi* flies with or without RU486-mediated transgene expression from day 5 onward, showing actin-rich rods (red channel, phalloidin) and nuclear DNA (blue channel, To-Pro-3). Scale bar is 20μm. (b) Immunostaining brains at 63x magnification from young (10-day-old) and aged (45-day-old) *elavGS*>*UAS-Fhos-RNAi* flies with or without RU486-mediated transgene expression from day 5 onward, showing actin-rich rods (red channel, phalloidin). Scale bar is 5 μm. (c) Quantification of actin-rich rods in brains as shown in (b). n = 5–6 flies per condition, as indicated. **p (d10 vs. aged RU−) = 0.0015, **p (Aged RU− vs. Aged RU+) = 0.0037, one-way ANOVA, Tukey’s multiple comparisons test. (d) Performance index in olfactory aversion training for 37-day-old *elavGS*>*UAS-Fhos-RNAi* flies with or without RU486-mediated transgene expression from day 5 onward, assessed by the number of flies avoiding a shock-associated scent versus the total number of flies participating in the assay. **p = 0.0026, ***p = 0.0001, one-way ANOVA, Tukey’s multiple comparisons test. (e) Performance index in olfactory aversion training for 37-day-old Canton S flies given vehicle (DMSO) or 10μM cytochalasin D as indicated from days 30–37 post eclosion, assessed by the number of flies avoiding a shock-associated scent versus the total number of flies participating in the assay. **p = 0.0023, ***p = 0.0001, one-way ANOVA, Tukey’s multiple comparisons test. (f) Con-ex feeding assay of 10-day-old *elavGS*>*UAS-Fhos-RNAi* flies with or without RU486-mediated transgene expression from day 5 onward. n = 6 vials of 10 flies per condition. ns = non significant, unpaired t-test. (g) Survival curve of *elavGS*>*UAS-Fhos-RNAi* flies with or without RU486-mediated transgene expression from day 5 onward. ***p = <0.0001, log-rank test. n = 118 RU− and 124 RU+ biologically independent animals. (h) Climbing pass rate of 58 day old *elavGS*>*UAS-Fhos-RNAi* flies with or without RU486-mediated transgene expression from day 5 onward. n = 180 biologically independent animals per condition measured in groups of 30. **p = 0.0079, unpaired t-test. (i) Climbing index as a measure of endurance of 45-day-old *elavGS*>*UAS-Fhos-RNAi* flies with or without RU486-mediated transgene expression from day 5 onward. n = 4 replicates of RU− and 4 replicates of RU+ with 100 biologically interdependent animals per replicate. *p = 0.0267, unpaired t-test. (j) Spontaneous physical activity of 44-day-old *elavGS*>*UAS-Fhos-RNAi* flies with or without RU486-mediated transgene expression from day 5 onward. n = 3 vials of 10 flies per condition. (k) Quantification of total activity per fly per hour from spontaneous activity graphs in (i). n = 3 vials of 10 biologically independent animals per condition. ***p < 0.0001, unpaired t-test. (k) Intestinal integrity during aging of *elavGS*>*UAS-Fhos-RNAi* flies with or without RU486-mediated transgene expression from day 5 onward. n = 8 vials of 30 biologically independent animals per vial on day 10. ***p < 0.0001 ; two-way ANOVA/ Šídák’s multiple comparisons test. RU486 or vehicle was provided in the media at a concentration fo 50 ug/ml in the indicated treatment groups. Data are presented as scatter plots overlaying mean values +/− SEM.

Since our observations found a correlation between aging and F-actin accumulation in the brain, we next decided to test if targeting neuronal F-actin polymerization genetically could affect organismal healthspan. We screened several genes related to actin and actin stabilization, including actin isoforms, actin-binding proteins (ABPs), and actin assembly factors, and assessed changes to age-related F-actin polymerization in the brain and to organismal lifespan. We found that neuronal knockdown of *Formin homology 2 domain containing ortholog* (*Fhos*), the *Drosophila* homolog of the FHOD sub-family of formins, had the most robust effect on these parameters. *Fhos* promotes actin nucleation for filament assembly ^40^. Using the pan-neuronal *Elav*-Gene-Switch (*elav*GS) driver line ^48^, we expressed *UAS-Fhos-RNAi* in adult fly neurons upon administration of the inducing agent RU486 in food. This system allows for cell-type specific induction of genetic constructs in a time- and dose-dependent manner. Flies in each experiment come from the same parental crosses and undergo identical developmental conditions. Phalloidin staining in aged brains revealed that neuronal *Fhos-RNAi* induction abrogated the actin-rich rods observed in aged control brains (Fig 2a,b,c). We found that midlife knockdown of the dominant actin isoform expressed in adult *Drosophila* neurons, *Act5c*, similarly reduced the number of actin-rich rods in the aged brain (Extended Data Fig 2a,b,c).

Actin plays an essential role in neuron polarity and, consequently, function ^49^. To assess physiological brain function, we tested associative learning and memory using olfaction aversion training ^50^. Briefly, flies were conditioned to associate a neutral odor (3-octanol, OCT) with a series of electric shocks. After one hour of rest, they were placed in a T-maze and allowed to choose between OCT and a second neutral odor (4-methylcyclohexanol). Young flies avoided the shock-associated OCT significantly more than aged flies (Fig 2c). Furthermore, aged flies expressing *Fhos-RNAi* in neurons showed a significantly better memory recall response than uninduced age-matched controls (Fig 2c). Remarkably, treating aged flies for one week with the actin destabilization drug cytochalasin D also significantly improved associative learning and memory (Fig 2d). Hence, reducing F-actin polymerization in aged brains, both genetically and pharmacologically, improved learning and memory in aged flies.

Changes in food intake can extend lifespan and delay age-related brain F-actin polymerization (Fig 1g,h,i). To test if neuronal expression of *Fhos-RNAi* in adult flies affected feeding behavior, we performed the consumption-excretion (“Con-Ex”) feeding assay ^51^. However, we observed no differences in food consumption and excretion (Fig 2e). With neuronal *Fhos*-RNAi induction correlating with a reduction in age-associated actin-rich rods in the brain and an improvement in memory, we next decided to test if organismal lifespan and additional parameters of health also improved. Remarkably, flies expressing *Fhos-RNAi* in neurons showed a dramatically increased lifespan compared to controls (Fig 2f). Similarly, midlife knockdown of *Act5c* in neurons significantly extended organismal lifespan (Extended Data Fig 2c). Furthermore, neuronal overexpression of *Twinstar* (*tsr),* the *Drosophila* homolog of *cofilin/ADF* (actin depolymerization factor) that plays a role in regulating actin cytoskeletal dynamics^52^, also extended *Drosophila* lifespan (Extended Data Fig 2d). In agreement, aged *elavGS*>*UAS-Fhos-RNAi* flies treated with RU486 showed improved locomotor activity (Fig 2g) and climbing endurance (Fig 2h) compared to vehicle-fed controls. Additionally, neuronal *Fhos-RNAi* induction conferred an increase in spontaneous daytime activity with no detectable nighttime restlessness in aged flies (Fig 2i,j).

Intestinal barrier dysfunction is an evolutionarily-conserved characteristic of aging associated with systemic inflammation, frailty, and mortality ^53^. To assess if neuronal knockdown of *Fhos* could prolong intestinal integrity, we performed the ‘Smurf assay’ ^54,55^. In agreement with prolonged lifespan and improved parameters of aging health, we observed a delay in gut leakiness in aged flies expressing *Fhos-RNAi* in neurons (Fig 2k).

To confirm that the effects of neuronal *Fhos* and *Act5c* knockdown were not an artifact of RNAi expression or RU486 administration, we generated flies expressing a construct of double-stranded RNA of *GFP* in neurons (*elavGS*>*UAS-dsGFP*). Importantly, induction of *dsGFP* did not affect age-associated brain F-actin polymerization (Extended Data Fig 2e,f), memory and learning (Extended Data Fig 2g), or feeding behavior (Extended Data Fig 2h). Furthermore, providing RU486 to *elavGS*>*UAS-dsGFP* flies did not extend organismal lifespan (Extended Data Fig 2i). Together, these findings indicate that neuronal knockdown of the actin nucleation gene *Fhos,* as well as *tsr* and *Act5c*, can significantly delay parameters of aging and extend organismal lifespan.

### Age-associated neuronal F-actin polymerization impairs brain autophagy

**Figure 3.**
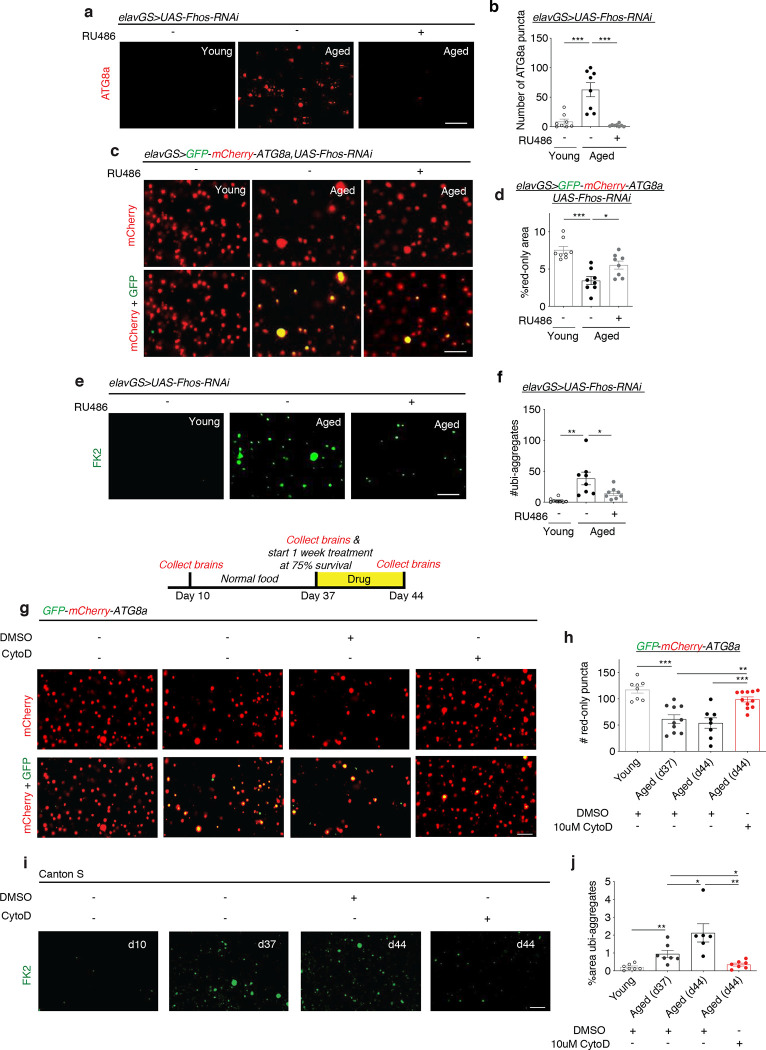
F-actin stabilization disables autophagy in the aged *Drosophila* brain. (a) Immunostaining of brains from young (10-day-old) and aged (45-day-old) *elavGS*>*UAS-Fhos-RNAi* flies with or without RU486-mediated transgene induction from day 5 onward, showing ATG8a levels (red channel, anti-ATG8a). Scale bar is 5 μm. (b) Quantification of Atg8a puncta in brain as shown in (a). n = 8 biologically independent animals, as indicated. ***p < 0.0001 (young vs. aged RU−), ***p < 0.0001(aged RU− vs. aged RU+); one-way ANOVA, Tukey’s multiple comparisons test. (c) GFP-mCherry-ATG8a of brains from 44-day-old e*lavGS*>*GFP-mCherry-ATG8a,UAS-Fhos-RNAi* flies. Images shown of GFP, mCherry, and merged GFP-mCherry channels. Scale bar is 5 μm. (d) Quantification of % area of autolysosomes (red-only puncta) in brains as shown in (c). n = 8 biologically independent animals per condition. *p = 0.0259; ***p < 0.0001; one-way ANOVA, Tukey’s multiple comparisons test. (e) Immunostaining of brains from young (10-day-old) and aged (44-day-old) *elavGS*>*UAS*-*Fhos-RNAi* flies with or without RU486-mediated transgene induction from day 5 onward, showing polyubiquitinated aggregates (green channel, anti-FK2). Scale bar is 5 μm. (f) Quantification of polyubiquitinated aggregates in brains as shown in (e). n = 8 biologically independent animals. *p=0.0331, **p=0.0016; one-way ANOVA/Tukey’s multiple comparisons test. (g) GFP-mCherry-ATG8a of brains from Canton S flies given vehicle (DMSO) or 10μM cytochalasin D at the indicated ages. Images shown of GFP, mCherry, and merged GFP-mCherry channels. Scale bar is 5 μm. (h) Quantification of the number of autolysosomes (red-only puncta) in brains as shown in (g). n = 8–10 biologically independent animals per condition, as indicated. ***p (young vs. aged d37 DMSO) < 0.0001; **p (aged d37 DMSO vs. aged d44 CytoD) = 0.0030; ***p (aged d44 DMSO vs. aged d44 CytoD) = 0.0007, one-way ANOVA, Tukey’s multiple comparisons test. (i) Immunostaining of brains from Canton S flies given vehicle (DMSO) or 10μM cytochalasin D at the indicated ages, showing polyubiquitinated aggregates (green channel, anti-FK2). Scale bar is 5 μm. (j) Quantification of % area of polyubiquitinated aggregates in brains as shown in (i). n = 6–7 biologically independent animals, as indicated. **p (young vs. aged d37 DMSO) = 0.0042, *p (aged d37 DMSO vs. aged d44 DMSO) = 0.0441, *p (aged d37 DMSO vs. aged d44 CytoD) = 0.0172, **p (aged d44 DMSO vs. aged d44 CytoD) = 0.0035; unpaired t-tests.

Actin dynamics are essential in the biogenesis and transportation of most cellular vesicles, including autophagosomes ^33^. Defects in autophagy is considered a primary hallmark of aging, resulting in impaired proteostasis and decreased organelle turnover ^20,23,30,56^. Furthermore, autophagy and vesicular trafficking defects have been identified in neurodegenerative diseases, including Alzheimer’s disease, Parkinson’s disease, and Huntington’s disease ^37,57^. With the changes observed to actin dynamics in the aging brain (Fig 1), we next sought to evaluate if interventions targeting age-associated F-actin polymerization would affect autophagy. Using endogenous LC3/ATG8 as a marker of autophagy, we found an age-related increase in autophagosomes reflective of reduced autophagic activity^53,58,59^ (Fig 3a,b). Neuronal-specific knockdown of actin nucleation factor *Fhos* resulted in a dramatic reduction in ATG8a accumulation, closely reflecting what was observed in young brains (Fig 3a,b). To further evaluate autophagic flux in the aging brain, we used a reporter line expressing GFP-mCherry-ATG8a (“ATG8a-tandem”) ubiquitously under the control of the endogenous ATG8 promoter ^60^. As autophagosomes fuse with lysosomes, GFP signal on the ATG8a tandem protein is quenched due to its sensitivity to low pH. Remaining mCherry-only foci indicate autolysosomal activity. When investigating the brains of young flies expressing ATG8a-tandem, we observed a striking density of red-only puncta and a near absence green signal. Conversely, aged brains showed a mixture of yellow and red-only puncta, with significantly fewer mCherry foci indicative of fewer autolysosomes (Fig 3c,d). These findings suggest that young brains display extensive autophagic flux that becomes stalled with age. When *Fhos-RNAi* was expressed in the neurons of adult ATG8a-tandem flies, we observed fewer yellow puncta and more red-only puncta in brains compared to aged controls, indicating recovery of autophagy (Fig 3c,d).

**Extended Data Figure 3.**
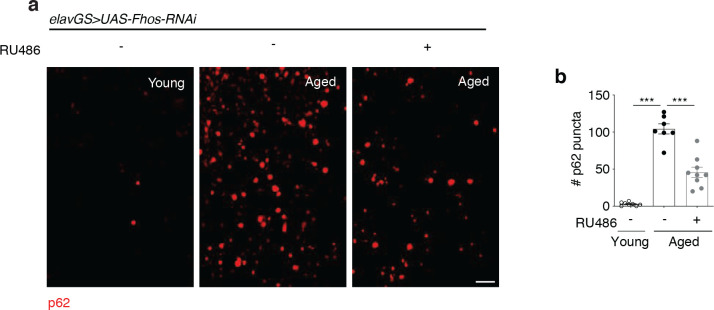
Genetic reduction of F-actin polymerization improves autophagic turnover of p62 in the aging *Drosophila* brain. (a) Immunostaining of brains from young (10-day-old) and aged (45-day-old) *elavGS*>*UAS-Fhos-RNAi* flies with or without RU486-mediated transgene induction from day 5 onward, showing p62 accumulation (red channel, anti-Ref(2)P/p62). Scale bar is 5 μm. (b) Quantification of p62 puncta in brain as shown in (a). n = 7–9 biologically independent animals, as indicated. ***p < 0.0001 (young vs. aged RU−), ***p < 0.0001 (aged RU− vs. aged RU+); one-way ANOVA, Tukey’s multiple comparisons test.

To complement our findings with ATG8, we tested additional readouts of protein homeostasis (proteostasis) and autophagy. Decline in proteostasis is another major cellular hallmark of aging ^20,56^, and it has been well characterized that aged tissues in *Drosophila* accumulate aggregates of ubiquinated proteins ^29,31,32,46,58,61,62^. We observed that neuronal *Fhos-RNAi* induction significantly reduced age-associated protein aggregates in the brain (Fig 3e,f). Ubiquinated proteins can be targeted for autophagic degradation by the adaptor protein p62/SQSTM1, with an accumulation of p62 indicating reduced turnover and breakdown similar to ATG8a accumulation ^53,58^. Aged brains showed significantly more p62 puncta compared to young brains, and neuronal knockdown of *Fhos* also reduced the accumulation of p62 in aged brains (Extended Data Fig 3a,b).

Next, we sought to test if a pharmacological intervention targeting actin polymerization could improve readouts of brain autophagy. We had earlier found that one-week treatment at midlife with cytochalasin D was sufficient to abrogate age-associated brain F-actin polymerization (Extended Data Fig 1j,k). Here, we tested the effect of cytochalasin D on flies expressing the ATG8a-tandem reporter. In a given cohort, brains were collected from young flies (10 days), aged flies before treatment (37 days), and aged flies after 1 week of treatment with cytochalasin D or vehicle from day 37 to day 44. Aged brains collected at day 37 and day 44 fed vehicle showed significantly fewer red-only ATG8a-tandem puncta compared to young brains, indicating a decline in autophagic flux. Remarkably, 1 week of cytochalasin D treatment in aged animals significantly increased red-only ATG8a-tandem foci compared to both day 44 and, critically, day 37 brains (Fig 3g,h). These findings indicate a reversal in age-related decline in autophagic flux when treating animals with a pharmacological inhibitor of actin polymerization. We next followed the same drug treatment paradigm while investigating proteostasis. Consistent with what was observed using the ATG8a-tandem reporter, treatment of wild-type flies for 1 week with cytochalasin D was sufficient to reverse age-related accumulation of ubiquinated proteins in the brain (Fig i,j). These findings imply that therapeutic targeting of age-associated actin polymerization may reverse both cellular hallmarks of brain aging and improve brain function.

### Neuronal F-actin polymerization in aged brains disrupts mitophagy

**Extended Data Figure 4.**
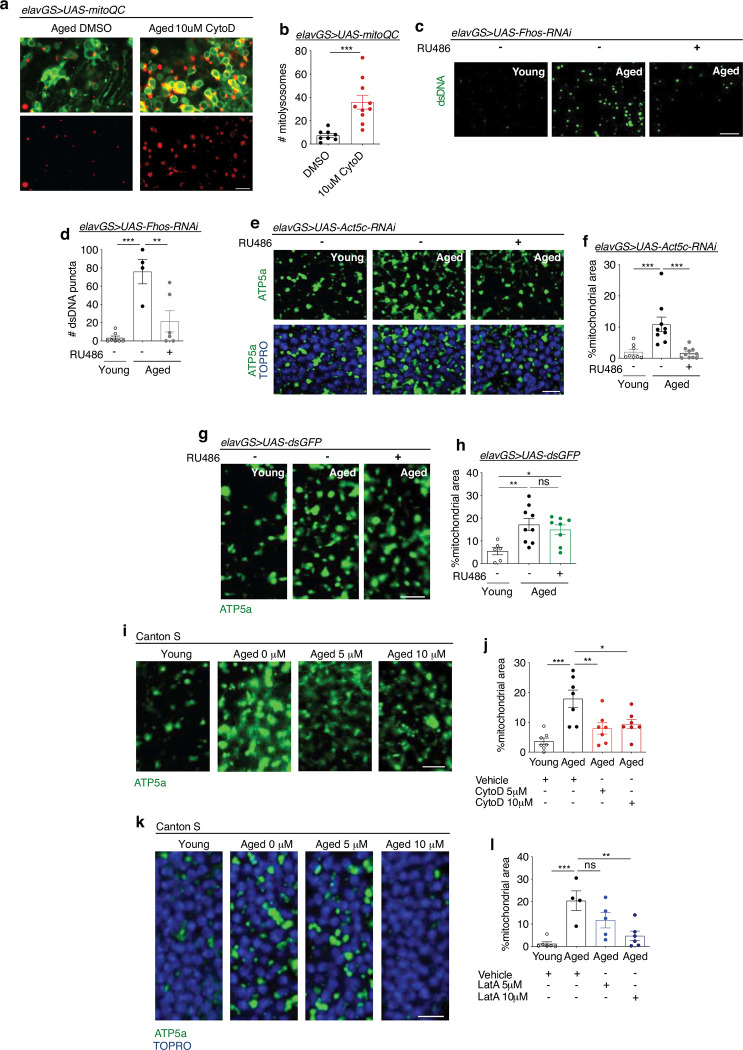
Genetic and pharmacological reduction of F-actin polymerization promotes mitophagy and reduces age-associated mitochondrial accumulation. (a) Mito-QC of brains from 44-day-old flies given vehicle (DMSO) or 10μM cytochalasin D from days 37–44 post eclosion. Images shown of merged GFP and mCherry along with punctate mCherry-only foci (from merged images where GFP has been quenched; mitolysosomes). Scale bar is 5 μm. (b) Quantification of mitolysosomes per 4mm^2^ brain area as shown in (a). n 8–10 biologically independent animals per condition, as indicated. ***p=0.0007, unpaired t-test. (c) Immunostaining of brains from young (10-day-old) and aged (45-day-old) *elavGS*>*UAS-Fhos-RNAi* flies with or without RU486-mediated transgene induction from day 5 onward, showing mitochondrial DNA accumulation (green channel, anti-dsDNA). Scale bar is 5 μm. (d) Quantification of dsDNA puncta in brain as shown in (c). n = 4–7 biologically independent animals, as indicated. **p=0.0038, ***p=0.0002; one-way ANOVA, Tukey’s multiple comparisons test. (e) Immunostaining of brains from young (10-day-old) and aged (45-day-old) *elavGS*>*UAS-Act5c-RNAi* flies with or without RU486-mediated transgene induction from day 5 onward, showing mitochondrial morphology (green channel, anti-ATP5a) and nuclear DNA (blue channel, stained with To-Pro-3). Scale bar is 5 μm. (f) Quantification of mitochondrial area in brains as shown in (c). n = 8–10 biologically independent animals per condition, as indicated. ns = non-significant, ***p (young vs. aged RU−) = 0.0009 ***p (aged RU− vs. aged RU+) = 0.0003; one-way ANOVA/Tukey’s multiple comparisons test. (g) Immunostaining of brains from young (10-day-old) and aged (45-day-old) *elavGS*>*UAS-dsGFP* flies with or without RU486-mediated transgene induction from day 5 onward, showing mitochondrial morphology (green channel, anti-ATP5a). Scale bar is 5 μm. (h) Quantification of mitochondrial area in brains as shown in (g). n = 6–9 biologically independent animals per condition, as indicated. ns = non-significant, *p = 0.0371, **p = 0.0077; one-way ANOVA/Tukey’s multiple comparisons test. (i) Immunostaining of brains from young (10-day-old) and aged (45-day-old) Canton S flies given vehicle (DMSO), 5 μM, or 10μM cytochalasin D at the indicated ages, showing mitochondrial morphology (green channel, anti-ATP5a). Scale bar is 5 μm. (j) Quantification of mitochondrial area in brains as shown in (e). n = 7 biologically independent animals per condition. *p = 0.0277, **p = 0.0083, ***p = 0.0002; one-way ANOVA/Tukey’s multiple comparisons test. (k) Staining of brains from young (10-day-old) and aged (45-day-old) Canton S flies given vehicle (DMSO), 5 μM, or 10μM Latrunculin A at the indicated ages, showing mitochondrial morphology (green channel, anti-ATP5a). Scale bar is 5 μm. (l) Quantification of mitochondrial area in brains as shown in (g). n = 4–7 biologically independent animals per condition, as indicated. p** = 0.0046, ***p = 0.0007, ns = non-significant; one-way ANOVA/Tukey’s multiple comparisons test.

**Figure 4.**
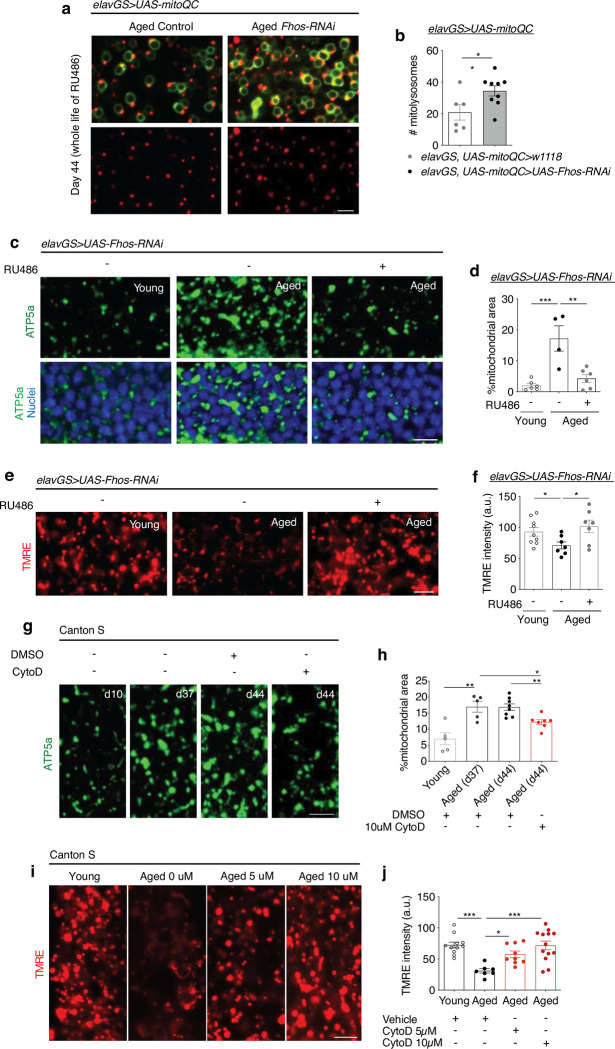
F-actin stabilization disables mitophagy in the aged *Drosophila* brain. (a) Mito-QC of brains from 44-day-old flies. Genotypes analyzed were *elavGS*>*UAS-mito-QC*, as a control, and *elavGS*>*UAS-mito-QC,UAS-Fhos-RNAi*. RU486-mediated transgenes were induced from day 5 onwards. Images shown of merged GFP and mCherry along with punctate mCherry-only foci (from merged images where GFP has been quenched; mitolysosomes). Scale bar is 5 μm. (b) Quantification of mitolysosomes per 4mm^2^ brain area as shown in (a). n 6–9 biologically independent animals per condition, as indicated. Area *p = 0.0305, unpaired t-test. (c) Immunostaining of brains from young (10-day-old) and aged (45-day-old) *elavGS*>*UAS*-*Fhos-RNAi* flies with or without RU486-mediated transgene induction from day 5 onward, showing mitochondrial morphology (green channel, anti-ATP5a) and nuclear DNA (blue channel, stained with To-Pro-3). Scale bar is 5 μm. (d) Quantification of mitochondrial area in brain as shown in (c). n = 4–6 biologically independent animals per condition, as indicated. **p = 0.0018, ***p = 0.0004; one-way ANOVA/Tukey’s multiple comparisons test. (e) Staining of brains from young (10-day-old) and aged (45-day-old) *elavGS*>*UAS-Fhos-RNAi* flies with or without RU486-mediated transgene induction from day 5 onward, showing TMRE fluorescence. Scale bar is 5 μm. (f) Quantification of mitochondrial membrane potential measured by TMRE staining as shown in (e). n = 7–9 biologically independent animals per condition, as indicated. *p (d10 vs. d45 RU−) = 0.0335, *p (d45 RU− vs. d45 RU+) = 0.0221, non-significant (n.s.); unpaired t-tests. (g) Immunostaining of brains from Canton S flies given vehicle (DMSO) or 10μM cytochalasin D at the indicated ages, showing mitochondrial morphology (green channel, anti-ATP5a). Scale bar is 5 μm. (h) Quantification of mitochondrial area in brain as shown in as shown in (i). n = 5–8 biologically independent animals, as indicated. **p (young vs. aged d37 DMSO) =0.0041, *p (aged d37 DMSO vs. aged d44 CytoD) = 0.0174, **p (aged d44 DMSO vs. aged d44 CytoD) = 0.0036; unpaired t-tests. (i) Staining of brains from young (10-day-old) and aged (45-day-old) Canton S flies given vehicle (DMSO), 5 μM, or 10μM cytochalasin D at the indicated ages, showing TMRE fluorescence. Scale bar is 5 μm. (j) Quantification of mitochondrial membrane potential measured by TMRE staining in brains as shown in as shown in (i). n = 7–13 biologically independent animals, as indicated. ***p (young vs. aged d44 DMSO) = 0.0003, *p (aged d44 DMSO vs. aged d44 5 μM CytoD) = 0.0353, ***p (aged d44 DMSO vs. aged d44 10 μM CytoD) = 0.0002; one-way ANOVA/Tukey’s multiple comparisons test.

One major role of autophagy is to mediate the turnover and clearance of damaged or superfluous mitochondria ^63^. Accordingly, we next sought to understand if age-associated F-actin polymerization in the brain interfered more specifically with mitophagy and mitochondrial function. To visualize mitophagic flux, we used the mito-QC reporter line that encodes a tandem GFP-mCherry fusion protein targeted to the outer mitochondria membrane ^64^. Mitochondria degraded in acidic lysosomes (mitolysosomes) appear as mCherry-only puncta as GFP is quenched. Consistent with our observations in autophagy, we found significantly more red-only foci in the aged brains of flies with neuronal knockdown of the F-actin nucleation factor *Fhos* compared to age-matched controls (Fig 4a,b). In agreement, midlife treatment of flies with cytochalasin D resulted in significantly more mitolysosomes in the brain compared to vehicle-fed controls (Extended Data Fig 4a,b). Consistent with reduced clearance of mitochondria, brains from aged *Drosophila* showed an accumulation of mitochondrial content compared to young adult controls (Fig 4c,d) as described ^29^. Neuronal knockdown of *Fhos* resulted in a significant reduction in mitochondrial content to an amount similar to that detected in young brains (Fig 4c,d). As an additional readout of brain mitochondrial content, we examined mitochondrial DNA (mtDNA) by IF by labeling non-nuclear double-stranded DNA. Aged brains showed significantly more mtDNA compared to young controls, and neuronal expression of *Fhos-RNAi* significantly reduced age-related mtDNA accumulation (Extended Data Fig 3c,d). In addition, adult-onset neuronal knockdown of *Act5c* also resulted in reduced mitochondrial content in aged brains (Extended Data Fig e,f). Control *elavGS*>*UAS-dsGFP* flies showed no change in brain mitochondrial content in aged flies treated with RU486 or vehicle (Extended Data Fig 4g,h). To assess function, we examined mitochondrial membrane potential using the potentiometric dye tetramethylrhodamine ethyl ester (TMRE). Aged brains showed a significant reduction in TMRE intensity compared to young controls, while targeting neuronal F-actin polymerization via *Fhos* knockdown resulted in recovery of mitochondrial membrane potential (Fig 4e,f). These data suggest that genetic targeting of F-actin polymerization in adult *Drosophila* brains results in increased mitophagy, reduced age-associated accumulation of mitochondria, and improved mitochondrial function.

Consistent with improved autophagy and mitophagy (Extended Data Fig 3g,h; 4a,b), treating middle-aged flies with cytochalasin D significantly reduced the accumulation of mitochondrial content in aged brains (Fig 4g,h). Notably, less brain mitochondrial content was detected at day 44, after 1 week of cytochalasin D treatment, compared to brains collected from flies of the same cohort at day 37 before treatment (Fig 4g,h), consistent with a reversal of this hallmark of brain aging. Mitochondrial content was reduced in aged brains at multiple concentrations of the drug (Extended Data Fig 4i,j). Treating aged flies for 1 week with an independent actin depolymerization agent, latrunculin A, also resulted in reduced mitochondrial content in aged brains in a dose-dependent manner (Extended Data Fig 4k,l). Importantly, TMRE staining revealed that pharmacologically depolymerizing age-associated F-actin by cytochalasin D significantly improved mitochondrial homeostasis in the aged brain (Fig 4i,j). Together, these findings indicate that genetic and pharmacological targeting of age-associated F-actin polymerization improves mitophagy and mitochondrial homeostasis in *Drosophila* brains.

### Neuronal reduction of F-actin polymerization slows aging via autophagy

**Figure 5.**
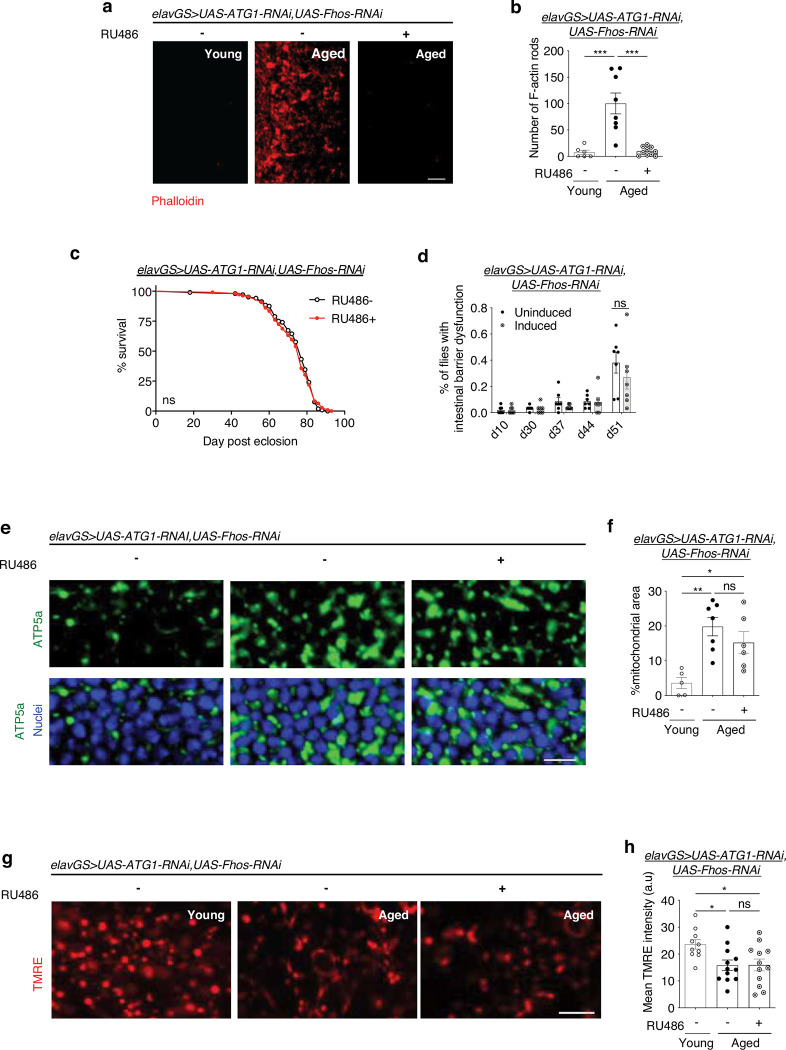
Neuronal reduction of F-actin polymerization slows aging via autophagy. (a) Immunostaining of optic lobes at 63x magnification from young (10-day-old) and aged (45-day-old) *elavGS*>*UAS-Fhos-RNAi,UAS-Atg1-RNAi* flies with or without RU486-mediated transgene expression from day 5 onward, showing actin-rich rods (red channel, Phalloidin). Scale bar is 5 μm. (b) Quantification of actin-rich rods in brains as shown in (a). n = 6–10 flies per condition, as indicated. ***p (young vs. aged RU−) = 0.0001, ***p (aged RU− vs. aged RU+) < 0.0001, one-way ANOVA/Tukey’s multiple comparisons test. (c) Survival curve of *elavGS*>*UAS-Fhos-RNAi,UAS-Atg1-RNAi* flies with or without RU486-mediated transgene expression from day 5 onward. ns = non-significant, log-rank test. n = 180 RU− and 180 RU+ biologically independent animals. (d) Intestinal integrity during aging of *elavGS*>*UAS-Fhos-RNAi,UAS-Atg1-RNAi* flies with or without RU486-mediated transgene expression from day 5 onward. n = 7 vials of 30 biologically independent animals per vial on day 10. ns = non-significant; two-way ANOVA/ Šídák’s multiple comparisons test. (e) Immunostaining of brains from young (10-day-old) and aged (45-day-old) *elavGS*>*UAS*-*Fhos-RNAi*,*UAS-Atg1-RNAi* flies with or without RU486-mediated transgene induction from day 5 onward, showing mitochondrial morphology (green channel, anti-ATP5a) and nuclear DNA (blue channel, stained with To-Pro-3). Scale bar is 5 μm. (f) Quantification of mitochondrial area in brain as shown in (e). n = 5–7 biologically independent animals per condition, as indicated. *p = 0.0277, **p = 0.0021, ns = non-significant; one-way ANOVA/Tukey’s multiple comparisons test. (g) Staining of brains from young (10-day-old) and aged (45-day-old) *elavGS*>*UAS-Fhos-RNAi,UAS-Atg1-RNAi* flies with or without RU486-mediated transgene induction from day 5 onward, showing TMRE fluorescence. Scale bar is 5 μm. (h) Quantification of mitochondrial membrane potential measured by TMRE staining as shown in (g). n = 10–12 biologically independent animals per condition, as indicated. *p (young vs. aged RU−) = 0.0296, * p (young vs. aged RU+) = 0.0318, non-significant (n.s.); one-way ANOVA/Tukey’s multiple comparisons test. RU486 or vehicle was provided in the media at a concentration of 50 ug/ml in the indicated treatment groups. Data are presented as scatter plots overlaying mean values +/− SEM.

Our findings indicate that age-associated F-actin polymerization in the brain disrupts autophagy, mitochondrial homeostasis, and proteostasis. Next, we set out to determine whether the beneficial effects of decreasing actin polymerization on brain and organismal aging are due to improved neuronal autophagy. First, we observed that *Fhos*-mediated modulation of F-actin levels during brain aging proceeds in an autophagy-independent manner. To block the autophagy pathway, we targeted the expression of *Atg1* (Autophagy-related 1, the *Drosophila* homolog of mammalian ULK1). This Ser/Thr protein kinase regulates the initiation of the formation of the autophagosome ^65^. Concomitant knockdown of neuronal *Atg1* and *Fhos* in *elavGS*>*UAS-Atg1-RNAi,UAS-Fhos-RNAi* flies resulted in reduced age-associated actin-rich rods in the brain (Fig 5a,b). In contrast to extended lifespan with neuronal knockdown of *Fhos* alone (Fig 2f), induced *elavGS*>*UAS-Atg1-RNAi,UAS-Fhos-RNAi* flies showed no difference in lifespan compared to vehicle-fed controls (Fig 5c). Consistent with this finding, we detected no difference in intestinal barrier integrity in flies with neuronal knockdown of both *Fhos* and *Atg1* (Fig 5d). Hence, although age-associated F-actin polymerization in the brain was reduced with neuronal *Fhos* knockdown, resulting health and lifespan benefits were dependent on autophagy.

Furthermore, concomitant knockdown of neuronal *Atg1* and *Fhos* in *elavGS*>*UAS-Atg1-RNAi,UAS-Fhos-RNAi* flies showed an age-associated accumulation of mitochondrial content in the brain (Fig 5e,f). Improvements to brain mitochondrial function that were found with disrupting age-associated F-actin polymerization were also lost with neuronal *Atg1* knockdown (Fig 5g,h). Together, these results indicate that improved mitochondrial homeostasis associated with reduced brain F-actin polymerization, like improvements to organismal health and lifespan, are dependent on autophagy. Cumulatively, these data are consistent with a model in which age-associated F-actin polymerization in *Drosophila* brains disrupts autophagy and, thereby, drives paradigms of brain and organismal aging.

## Discussion

Actin filaments show a loss of stability and deterioration in aged yeast cells ^66^ and in multiple non-neuronal tissues of aged *C. elegans* ^5^. In contrast, we find a striking age-related increase in F-actin and actin-containing rods in the fly brain, which contribute to brain aging and drive organismal health decline. Our findings are consistent with studies showing excess actin stabilization drives neurotoxicity in models of Alzheimer’s disease (AD) and related tauopathies ^15,16^ and Parkinson’s disease (PD) ^17,67^. As advanced age is a major risk factor for sporadic forms of both AD and PD, actin hyperstabilization may be a shared pathogenic mechanism of age-onset neurodegeneration. Future work will be required to determine the precise cellular mechanisms that lead to excess F-actin and the formation of actin-rich rods in the aged brain. The formation of rodlike inclusions (rods) composed of actin and actin assembly-regulatory proteins has been shown in cultured neurons in response to various stressors, including oxidative stress and mitochondrial energetic impairments ^68–70^. As mitochondrial dysfunction is a major hallmark of brain aging, it is reasonable to suggest that there could be a ‘vicious cycle’ whereby mitochondrial dysfunction and actin hyperstabilization drive brain aging. In addition, it has been shown that *de novo* actin polymerization is required to form model Hirano bodies ^71^. In the context of our study, it is interesting to speculate that actin nucleation is driving excess actin stabilization, and actin-rich rod formation, in the aged brain. The most pronounced phenotype that we observe with respect to lifespan extension is mediated by neuron-specific RNAi of *Fhos,* which shares the capacity of other formins to nucleate and bundle actin filaments ^40^. We also observe increased expression of actin transcripts in the aged brain. Hence, it is possible that the overall increase in F-actin in the aged brain is due to a combination of increased actin expression and hyperstabilization. Regardless of the mechanisms at play, our findings reveal F-actin accumulation to be an important hallmark of brain aging, which should be considered in the context of existing hallmarks of aging ^21^.

One of the key hallmarks of brain aging which drives age-onset pathology is disabled autophagy ^21,72^ given its demonstrated capacity to remove aggregated proteins and damaged organelles linked to neurodegenerative disease. Hence, identifying interventions that restore autophagy in the aged brain is a promising therapeutic avenue, but that depends on the step in the process that is impaired and the nature of the impairment. Unfortunately, our understanding of the nature of autophagy impairments in the aged brain and in most cases of neurodegeneration is limited, making it uncertain whether the same type of intervention is likely to work in all diseases and at all stages of those diseases. Indeed, it has been proposed that dysfunctional autophagy in aged animals, linked to blockage of autophagy at a late stage, may contribute to age-onset health decline ^30,73^. Hence, it is possible that interventions that induce early stages of autophagy may not prove effective when targeted to aged animals. In this study, we show that inhibiting F-actin polymerization in aged neurons prevents the age-related loss of autophagic activity in the brain. Moreover, we show that treating middle-aged flies with an actin polymerization inhibitor, cytochalasin D, restores brain autophagy to pre-treatment levels and leads to lower amounts of protein aggregates and mitochondrial content in the brain than before the treatment began. Interestingly, our data indicate that the accumulation of F-actin in the aged brain is not due to impaired autophagy. We show that the ability of neuronal *Fhos* inhibition to prevent F-actin accumulation in the aged brain is autophagy-independent. However, reducing F-actin levels in the aged brain while disrupting autophagy fails to improve mitochondrial homeostasis in the aged brain or prolong healthspan. Hence, our current working hypothesis is that disrupting actin polymerization in aging neurons prolongs healthspan via improvements in brain autophagy.

The functional significance of abnormal F-actin accumulation in cellular health and disease is highlighted by clinical data studying neurodegenerative diseases. There is an emerging consensus that F-actin containing intracellular inclusions disrupt neuronal function and are a likely cause of synaptic loss without neuronal loss, as occurs early in dementias ^74,75^. Here, we show that disrupting actin polymerization in the brains of middle-aged animals robustly improves a well-established paradigm of olfactory learning ^50,76^; the ability of flies to associate an odor with an aversive stimulus. Our findings reveal that inhibiting actin polymerization in aged animals can slow or even reverse aspects of brain aging. It is important to consider, however, that it is unlikely that disrupting actin polymerization in every cell and tissue-type would promote organismal health. It is likely that actin polymerization is essential to cell and tissue homeostasis in numerous contexts. Hence, in order to translate these findings to benefit human health, future work could focus upon identifying cell-type and tissue-specific approaches to inhibit actin polymerization in aged organisms.

## Methods

### Fly stocks

The fly strain *Elav–GeneSwitc*h (*ElavGS*) was provided by H. Keshishian (Yale University, New Haven, CT, USA). GFP-mCherry-Atg8a was provided by Eric Baehrecke (University of Massachusetts Medical School, Worcester, MA, USA). *UAS-mito-QC* was provided by Alexander J. Whitworth (University of Cambridge, UK). *UAS-Fhos-RNAi* (31400, 51391)*, UAS*-*tsr* (20665)*, UAS-Act5c-GFP* (7309), *UAS-Act42a-GFP* (9252), *UAS-GFP-dsRNA* (9330) were acquired from the Bloomington Stock Center. *UAS-Act5c-RNAi* (101438) and *UAS-Atg1-RNAi* (16133) lines were received from Vienna *Drosophila* RNAi Center (VDRC).

### Fly Husbandry and Lifespan Analysis

Flies were maintained in vials containing cornmeal medium (1% agar, 3% yeast, 1.9% sucrose, 3.8% dextrose, 9.1% cornmeal, 1.1% acid mix, and 1.5% methylparaben, all concentrations given in wt/vol). Flies were collected under light anesthesia by nitrogen gas and housed at a starting density of 30 fertilized female flies per vial. All flies were kept in a humidified, temperature-controlled incubator with a 12h:12h dark:light cycle at 25 °C. RU486 was dissolved in ethanol and administered in the media as indicated while preparing food. Cytochalasin D (Sigma-Aldrich C2618) and Latrunculin A (Sigma-Aldrich 428026), or DMSO vehicle control, were mixed in media as indicated. Flies were flipped to fresh vials every 2–3 days and scored for death.

### Immunostaining and Image analysis

For brain immunostaining, flies were fixed in 3.7% formaldehyde in phosphate buffered saline (PBS) for 20 min. After fixation, brains were dissected and fixed again for 5 min. Samples were then rinsed 3 times for 10 min with PBST and blocked in 3% BSA in PBST (PBST-BSA) for 1 hour. Primary antibodies were diluted in PBST-BSA and incubated overnight at 4°C. Primary antibodies used were: mouse-anti-ATP5a 1:250 (15H4C4, abcam); mouse anti-actin 1:50 (JLA20, DSHB), mouse-anti-FK2 1:250 (BML-PW8810–0500, ENZO); rabbit-anti-atg8a 1:250 (Rana et al., 2017). mouse-anti-dsDNA 1:250 (ab27156, abcam). Samples were then rinsed 3 times in PBST for 10 min. and incubated with the secondary antibodies and/or stained at room temperature for 3 hours. Secondary antibodies and stains used were: anti-rabbit or anti-mouse AlexaFluor-488 1:500 (A-11001 or A-11008, Thermo Fisher Scientific); anti-rabbit or anti-mouse AlexaFluor-568 1:500 (A-11031 or A-11036, Thermo Fisher Scientific); To-Pro-3 DNA 1:500 (T3605, Thermo Fisher Scientific; phalloidin AlexaFlour-568 1:250 (A12380, Thermo Fisher Scientific). Finally, samples were rinsed 3 times with PBST for 10 min and mounted in Vectashield Mounting Medium (Vector Lab). Images were taken using Zeiss LSM780 or LSM880 confocal microscope and analyzed with ImageJ software to measure intensity, area, count, and/or sizes of stained structures.

### TMRE staining

Flies were anesthetized and dissected in cold Drosophila Schneider’s Medium (DSM). Brains were incubated in TMRE staining solution (100nm TMRE (T669, Thermo Fisher Scientific) in DSM) for 12 min at room temperature. After staining, samples were rinsed once in wash solution (25nm TMRE in DSM) for 30 seconds before being mounted in wash solution. Images were acquired using a Zeiss LSM880 confocal microscope and TMRE intensity was quantified using ImageJ software.

### GFP-mCherry-Atg8a tandem and Mito-QC staining

Flies were anesthetized and dissected in cold Drosophila Schneider’s Medium (DSM). Brains were mounted in DSM solution. Images were acquired on a Zeiss LSM780 or LSM880 confocal microscope and autolysosomes or mitolysosomes (mCherry-only foci) were quantified using ImageJ software.

### Olfactory training

Aversion training was performed as described in ^50^ using a system from MazeEngineers (Conduct Science). Briefly, flies were exposed to a neutral odor (3-octanol) by air pump in a training chamber for one minute under low red light conditions. They were then exposed to the odor in a series of twelve 60-V shocks for 1.25 seconds followed by rest for 3.75 seconds for a total of one minute. Flies recovered for one hour before being placed in a T-maze with the trained scent on one side and a second neutral scent (4-methylcyclohexanol) on the other side of the maze. After two minutes of exploration under dim red light conditions, flies in either chamber of the maze were counted.

### Intestinal barrier dysfunction (Smurf) assay

Intestinal integrity assays were performed as previously described ^55^. Flies were aged to the indicated time points with standard RU− or RU+ food as indicated. To conduct the “Smurf” assay, flies were then transferred to new vials containing standard medium with 2.5% wt/vol F&D blue dye # 1 (SPS Alfachem) for 16 hours. The number of flies per vial with dye coloration outside the gut (Smurf flies) were then tallied and quantified.

### Climbing activity assay

In negative geotaxis assays, flies were gently tapped to the bottom of 10 cm vials. After 10 seconds, the number of flies that climbed above 5 cm were recorded. For forced climbing assays, 100 adult flies from each treatment group were placed in 200 ml glass cylinders. The cylinders were tapped quickly and the flies were allowed to settle for 2 minutes. This step was repeated nine times. 1 minute after the final tap, the number of flies in the upper, middle, and lower third of the cylinder was recorded.

### Spontaneous physical activity assay

Vials containing 10 adult flies were placed inside a *Drosophila* activity monitor (TriKinetics). Movements were recorded continuously under normal culturing conditions for 36 hours on a 12h:12h dark:light cycle. Graphs represent mean activity per fly per hour and the scatterplot shows spontaneous activity per fly during a 12h:12h dark:light cycle. Triplicate samples were used for each activity measurement

### Consumption-Excretion (Con-Ex) assay

Con-EX assays were performed as previously described ^51^. Adult flies were transferred to new empty vials (10 flies per vial with a total of 6 vials) and fed from feeder caps containing standard medium with 2.5% wt/vol F&D blue dye # 1 for 20h at 25 °C. Feeder caps were discarded at the conclusion of feeding. For checking internal (consumed) dye, flies were homogenized in 500 ul of ddH_2_O and pellet debris were removed by centrifugation. The dye excreted by the flies on the walls of the vials was collected by adding 1ml of ddH2O to each vial and vortexing. Samples were quantified using an Epoch BioTek microplate reader and compared to a serially diluted standard.

### Quantitative real-time PCR.

Total RNA was extracted from samples using TRIzol reagent (Invitrogen) following manufacturer protocols. Samples were treated with DNAse before cDNA synthesis was performed using the First Strand cDNA Synthesis Kit from Fermentas. qPCR was performed using Power SYBR Green master mix (Applied Biosystems) on a BioRad Real-Time PCR system. Cycling conditions were as follows: 95°C for 10 min; 95°C for 15 s then 60°C for 60 s, cycled 40 times. GAPDH or RPL32, as indicated, were used as reference genes to normalize. BioRad CFX Manager ver. 3.1 was used to collect and analyze qRT-PCR data. Primer sequences used are as follows:

GAPDH, GCGGTAGAATGGGGTGAGAC and TGAAGAGCGAAAACAGTAGC

RPL32, GACCATCCGCCCAGCATAC and CGGCGACGCACTCTGTT

Act5c, AGGCCAACCGTGAGAAGATG and GGGGAAGGGCATAACCCTC

Act42a, ATGGTAGGAATGGGACAAAAGGA and CTCAGTAAGCAAGACGGGGTG

### Enzyme-linked Immunosorbent Assays (ELISAs)

ELISA specific for F-actin (MyBiosource) was used according to manufacturer protocol. Five fly heads were homogenized in 30 μl of homogenizing F-actin stabilization buffer (Cytoskeleton). The sample was diluted in sample diluent at a ratio of 1:20 before being loaded to a microplate that was pre-coated with an antibody specific for F-actin. A biotinylated secondary antibody was added to the microplate and subsequently incubated with HRP-avidin followed by peroxidase substrate. Concentrations of F-actin were determined using a Epoch BioTek microplate reader and compared to a serially diluted standard provided by the manufacturer.

### Statistics

GraphPad Prism 9 (GraphPad Software, La Jolla, CA, USA) was used to perform statistical analysis and graphically display data. Significance is expressed as *p* values as determined by two-tailed, unpaired, parametric, or non-parametric tests as indicated in figure legends. When comparing two groups, unpaired t-tests were used when data met criteria for parametric analysis and Mann-Whitney tests were used for non-parametric analysis. To compare more than two groups when parametric tests were appropriate, one-way ANOVAs with Tukey’s multiple comparisons tests were performed. To compare more than two groups sampled from a Gaussian distribution without assuming equal variances, Welch and Brown-Forsythe ANOVAs were used. To analyze more than two groups when data did not meet requirements for parametric tests, Kruskal-Wallis tests with Dunn’s multiple comparisons post hoc tests were used. When performing grouped analyses with multiple comparisons, two-way ANOVAs with Šídák’s multiple comparisons test were performed. Bar graphs depict mean ± standard error of the mean (SEM). The number (n) of biological samples used in each experiment can be found in figure legends. Log-rank (Mantel-Cox) tests were used to compare survival curves. No statistical methods were used to pre-determine sample sizes but our sample sizes are similar to those reported in previous publications ^58,62^. Blinding was performed when possible, specifically when conducting microscopy for TMRE, ATG8a-tandem, and mitoQC. Blinding was not always possible during experimental setup due to the need to carefully document the genotypes of flies when generating crosses or to track groups assigned RU486 vs. vehicle throughout lifespans. All experiments were conducted under the same conditions, and control and experimental samples were treated equally and in parallel to exclude bias. Additionally, all images were taken in the same location and depth in each tissue type. Parents of experimental flies were randomly grouped into mating vials with 10 virgin females to 7 mature males. Upon eclosion, experimental flies were randomly assigned to mating bottles (10 vials per bottle) for 3 days. These bottles were then sorted into vials containing 30 mated females each before evenly distributing these vials assigned randomly into treatment and control groups. No animals or data points were excluded from the analyses. The difference between two groups was defined as statistically significant for the following *p* values: *p<0.05, **p<0.01, ***p< 0.001 (and non-significant when *p*>0.05).

## Extended Data

## Data Availability

All data generated or analyzed during this study are included in the figures and text with representative images accompanying quantified results where applicable unless otherwise noted. Further information is available from the corresponding author upon reasonable request.
